# Report of Mortality in Buffalo for the Month of July, 1856

**Published:** 1856-09

**Authors:** C. L. Dayton

**Affiliations:** Health Physician


ART. XI.—*Report of Mortality in Buffalo for the month of July*, 1856. By C. L. DAYTON, M. D., Health Physician.

| DISEASES. | No. | Males. | Females. |
|---|---|---|---|
| Accidens, | 3 | 3 | |
| Asphyxia Immersorum, | 14 | 11 | 3 |
| Apoplexia, | 1 | 1 | |
| " a Potu, | 1 | 1 | |
| Cyanosis, | 1 | 1 | |
| Cholera Infantum, | 9 | 1 | 8 |
| Cirrhosis Hepatis, | 2 | 1 | 1 |
| Cynanche Trachealis, | 1 | 1 | |
| Cholera Morbus, | 2 | 1 | 1 |
| Diarrhœa, | 12 | 5 | 7 |
| Dysentery, | 3 | 1 | 2 |
| Dentitio, | 2 | 2 | |
| Dissipatio, | 1 | 1 | |
| Delirium Tremens, | 2 | 2 | |
| Debilitas Senilis, | 1 | | 1 |
| Enteritis, | 2 | | 2 |
| Eclampsia, | 17 | 10 | 7 |
| Febris Scarlatina, | 2 | 1 | 1 |
| " Typhus, | 2 | 1 | 1 |
| " Typhoid, | 4 | 4 | |
| " Ignotus, | 2 | 1 | 1 |
| Fracture Cranii, | 1 | 1 | |
| Gastritis, | 1 | | 1 |
| Hydrocephalus, | 6 | 5 | |
| Hydrothorax, | 1 | 1 | |
| Hydrops, | 2 | 2 | |
| Ignotus, | 22 | 12 | 9 |
| Icterus, | 2 | 1 | 1 |
| Ictero Duodenitis, | 1 | | 1 |
| Marasmus, | 1 | | 1 |
| Mania Puerperarum, | 1 | | 1 |
| Narcosis, | 1 | | 1 |
| Pneumonitis, | 3 | 2 | 1 |
| Pneumo Pericarditis, | 1 | 1 | |
| Peritonitis, | 2 | | 2 |
| Phrenitis, | 1 | 1 | |
| Pleuritis Chronica, | 1 | | 1 |
| Parturitio, | 1 | | 1 |
| Premature Birth, | 1 | 1 | |
| Rubeola, | 1 | | 1 |
| Rheumatism | 1 | | 1 |
| Summer Complaint, | 42 | 23 | 16 |
| Still-born, | 3 | 1 | 2 |
| Scirrhus Uterina, | 1 | | 1 |
| Scrophula, | 1 | 1 | |
| Tonsilitis Asphyxiatum, | 1 | | 1 |
| Tuberculosis Pulmonalis, | 7 | 2 | 5 |
| " Intestinalis, | 1 | | 1 |
| Total, | 191 | | |

SEXES.

| | |
|---|---|
| Males, | 103 |
| Females, | 83 |
| Sex not given, | 5 |
| Total, | 191 |

AGES.

| | | | |
|---|---|---|---|
| Still-born, | 3 | Between 10 years and 20 years, | 6 |
| 1 day, | 2 | " 20 " " 30 " | 15 |
| Between 1 day and 30 days, | 19 | " 30 " " 40 " | 11 |
| " 1 month and 6 months, | 44 | " 40 " " 50 " | 11 |
| " 6 months and 12 " | 28 | " 50 " " 60 " | 7 |
| " 1 year " 3 years, | 33 | " 60 " " 70 " | 5 |
| " 3 " " 5 " | 1 | " 70 " " 80 " | 0 |
| " 5 " " 10 " | 6 | " 80 " " 90 " | 0 |
| Total number under 10 years, | 136 | Total number over 10 years, | 55 |
| | | Total, | 191 |

NATIVITIES.

| | | | |
|---|---|---|---|
| Americans, | 100 | Prussian, | 0 |
| German, | 38 | French, | 1 |
| Irish, | 23 | Scotch, | 1 |
| English, | 4 | Holland, | 3 |
| Canadian, | 0 | Country not given, | 21 |
| | | Total, | 191 |

